# The effect of shock duration on trauma-induced coagulopathy in a murine model

**DOI:** 10.1186/s40635-021-00428-1

**Published:** 2022-01-07

**Authors:** Pieter H. Sloos, M. Adrie W. Maas, Markus W. Hollmann, Nicole P. Juffermans, Derek J. B. Kleinveld

**Affiliations:** 1 Department of Intensive Care Medicine, Amsterdam UMC Location AMC, Amsterdam, The Netherlands; 2 Laboratory of Experimental Intensive Care and Anesthesiology, Amsterdam UMC Location AMC, Amsterdam, The Netherlands; 3 Department of Anaesthesiology, Amsterdam UMC Location AMC, Amsterdam, The Netherlands; 4 Department of Intensive Care Medicine, Onze Lieve Vrouwe Gasthuis, Amsterdam, The Netherlands; 5 Department of Intensive Care Medicine, Erasmus MC, Rotterdam, The Netherlands

**Keywords:** Trauma, Coagulopathy, Shock

## Abstract

**Background:**

Trauma-induced coagulopathy (TIC) is a life-threatening condition associated with high morbidity and mortality. TIC can present with different coagulation defects. In this study, the aim was to determine the effect of shock duration on TIC characteristics. We hypothesized that longer duration of shock leads to a more hypocoagulable rotational thromboelastometry (ROTEM) profile compared to a shorter duration of shock.

**Methods:**

Male B57BL/6J(c) mice (*n* = 5–10 per group) were sedated and mechanically ventilated. Trauma was induced by bilateral lower limb fractures and crush injuries to the liver and small intestine. Shock was induced by blood withdrawals until a mean arterial pressure of 25–30 mmHg was achieved. Groups reflected trauma and shock for 30 min (TS30) and trauma and shock for 90 min (TS90). Control groups included ventilation only (V90) and trauma only (T90).

**Results:**

Mice in the TS90 group had significantly increased base deficit compared to the V90 group. Mortality was 10% in the TS30 group and 30% in the TS90 group. ROTEM profile was more hypocoagulable, as shown by significantly lower maximum clot firmness (MCF) in the TS30 group (43.5 [37.5–46.8] mm) compared to the TS90 group (52.0 [47.0–53.0] mm, *p* = 0.04). ROTEM clotting time and parameters of clot build-up did not significantly differ between groups.

**Conclusions:**

TIC characteristics change with shock duration. Contrary to the hypothesis, a shorter duration of shock was associated with decreased maximum clotting amplitudes compared to a longer duration of shock. The effect of shock duration on TIC should be further assessed in trauma patients.

**Supplementary Information:**

The online version contains supplementary material available at 10.1186/s40635-021-00428-1.

## Background

Haemorrhage after trauma is a leading cause of preventable mortality worldwide [1]. Haemorrhaging trauma patients frequently present with trauma-induced coagulopathy (TIC), which is associated with increased transfusion requirements and mortality [2, 3]. TIC can manifest with hypocoagulable, hypercoagulable or mixed characteristics [4]. A hypocoagulable state is often present early after trauma and is characterised by coagulation factor depletion, dysfunctional platelets and hyperfibrinolysis [5–7]. These components lead to an unstable clot formation and reduced clot strength, resulting in a disability to control the ongoing haemorrhage [8]. Hypocoagulable profiles can shift towards a more hypercoagulable state, characterised by increased thrombin generation, platelet activation and fibrinolytic shutdown [9–11]. Hypercoagulability often develops later on after trauma, but can also be present as early as minutes to hours after trauma [12–14]. The mechanisms underlying TIC characteristics are largely unknown. Shock is thought to play a major role in hypocoagulation, and its presence is associated with adverse outcomes [2]. The duration of shock differs between trauma patients; however, it is currently unknown how the duration of shock influences TIC [15]. Unravelling the modulatory effects of shock duration on TIC characteristics has implications for the timing of treatment strategies aimed at reducing TIC. In this study the aim was to compare the effects of shock duration on TIC. We hypothesised that longer duration of shock, is associated with a more hypocoagulable profile compared to short duration of shock.

## Methods

### Ethics

Experiments were performed with approval of the Institutional Animal Care and Use Committee of the Amsterdam UMC, location AMC. Procedures were performed in accordance with the European Parliament directive (2010/63/EU) and the Dutch national law the Experiments on Animals Act (Wod, 2014). Male B57BL/6J (c) mice were ordered from Charles River (USA) and housed in the on-site animal housing facility 7 days before the experiment. Animals had excess to food (Teklad global 16% protein, Envigo, USA) and water ad libitum with regular 12-h day–night cycle. All mice were 8 weeks during the experiment with a weight of 20–30 g.

### Animal model

Mice were sedated with 3–4% isoflurane (Isoflutek, Karizoo, Spain) and injected intraperitoneally with 0.06 mg/kg fentanyl (Hameln, Germany). During the tracheostomy procedure, mice received mask ventilation (2% isoflurane, 50% Fio2). After tracheostomy, mice were mechanically ventilated for the remaining part of the experiment (VentElite, Harvard Apparatus, USA) with tidal volumes of 7 ml/kg, respiratory rate of 160 breaths per minute and an inspiratory/expiratory ratio of 1:1.5 and FiO_2_ of 40%. Mice remained anaesthetized throughout the entire experiment with 1–2% isoflurane. Anaesthetic depth was deemed adequate if there was no reaction to painful stimulus, observed by absence of pedal reflex and/or change in blood pressure. An inspiratory sigh of 20% was performed every 30 min as recruitment manoeuvre.

The right carotid artery (arterial blood pressure monitoring) and jugular vein were cannulated after which mice received intravenous fentanyl 0.12 mg/kg (Pump 11 Pico Plus Elite, Harvard Apparatus, USA) and 20 ml/kg maintenance fluids consisting of Ringer’s lactate supplemented with 15.3 mM glucose and 2 mM sodium bicarbonate (BBraun Perfuser, Germany). Temperature was monitored continuously with a rectal thermometer and kept at 37 °C using a heated table and heat lamp.

Mice were randomised to one of the following groups: 90 min ventilation (V90), trauma + 90 min ventilation (T90), trauma + 30 min shock (TS30), trauma + 90 min shock (TS90). Trauma consisted of bilateral lower limb fractures using two haemostatic forceps. Median laparotomy was performed to induce crush injury by clamping the small intestine distally of Treitz ligament five times for 2 s. Liver injury was achieved by clamping 1 cm of the right lobe for 2 s. Following trauma, the abdomen was closed.

In group TS30 and TS90, preceding trauma, 200 µl blood was drawn through the carotid artery. Following trauma, additional blood was drawn to achieve a target mean arterial pressure (MAP) of 25–30 mmHg. After this blood pressure was reached (± 20 min after cannulation), no additional blood was withdrawn. Temperature was passively lowered to 35 °C in the TS30 and TS90 group and maintained at this temperature throughout the experiment. At the end of the experiment, blood was drawn though the carotid artery or via heart puncture. An overview of the experimental setup is shown in Additional file 1: Fig. S1.

### Blood sampling

At the end of the experiment, the first 50 µl blood/saline was discarded, after which 200 µl blood was collected in a heparin coated syringe for arterial blood gas analysis (RAPIDPoint 500, Siemens, Germany). The next 50 µl blood was discarded to prevent heparin contamination and the remaining blood was collected in 3.2% sodium citrate (1:9 ratio). A part of the collected citrated whole blood was used for rotational thromboelastometry (ROTEM, Werfen, Spain). The remaining citrated blood was centrifuged twice at 2500*g* for 15 min at 4 °C (centrifuge 5430R; rotor FA-45-30-11, Eppendorf, Hamburg, Germany) and frozen in liquid nitrogen before storage at − 80 °C until further analysis.

### Rotational thromboelastometry

The ex-tem assay measures the tissue factor pathway by addition of 7 µl ex-tem reagent (containing tissue factor) and 7 µl star-tem (containing phospholipids and calcium) to 105 µl citrated whole blood sample. EXTEM was performed using ROTEM minicups (Werfen, Spain), according to manufacturer’s guidelines. Clotting time (CT) measures the initiation of clot formation, the alpha (α) angle represents the angle between the baseline and the tangent through the 2 mm point. Maximum clot firmness (MCF) depicts the maximum clot strength and maximum lysis (ML) shows the maximum lysis in percentage detected during the 90 min run time.

### Enzyme-linked immunosorbent assay (ELISA)

d-dimer levels were measured using ELISA according to manufacturer’s instructions (Elabscience, USA).

### Organ wet/dry ratios

The left lung, part of the liver, and left kidney were collected and wet weight was determined at after the experiment. After drying the organs at 37 °C for 7 days, they were weighted again to determine wet/dry ratios.

### Sample size analysis

Based on pilot experiments, we determined 8 mice were needed to detect a 10 mm difference in ROTEM MCF between TS30 and TS90 with a common standard deviation (SD) of 5 mm (α = 0.05 and a power of 80%). To account for 20% mortality in our model, 10 mice were used in the TS30 and TS90 group.

### Statistical analysis

Data were analysed using SPSS version 25.0 (IBM, New York, USA). Graphs were made using GraphPad Prism version 9.0 (San Diego, USA). The histograms of all parameters were visually inspected for distribution. Parametric data were presented as mean with standard deviation (SD). Non-parametric data were presented as median with interquartile range (IQR) and analysed with Kruskal–Wallis test with post hoc Dunn’s test, corrected for multiple testing. Binominal data were analysed with the Fisher’s exact test. A *p* value of less than 0.05 was considered to be statistically significant.

## Results

### Trauma, shock and mortality

**Fig. 1:**
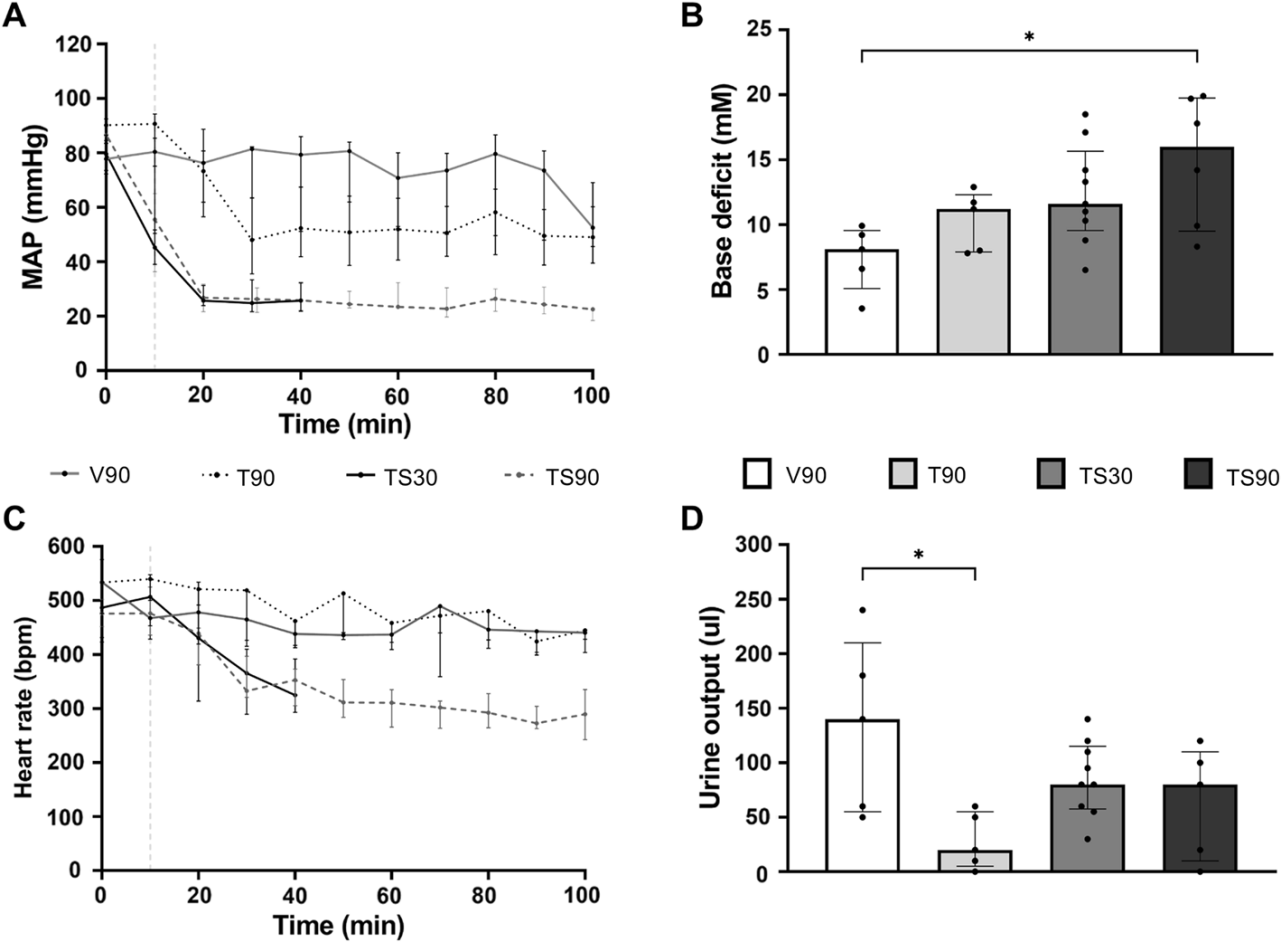
Haemodynamic parameters during different shock durations. Data are presented as median with interquartile range. **A** Mean arterial pressure. **B** base deficit. **C** Heart rate. **D** Urine output. V90 = 90 min ventilation, T90 = trauma + 90 min ventilation, TS30 = trauma + 30 min shock, TS30 = trauma + 90 min shock. *p < 0.05 between groups

The amount of blood withdrawn to reach the predefined MAP target was similar between groups: 330 µl (± 40 µl) in TS30 group and: 350 µl (± 60 µl) in the TS90 group. TS90 resulted in increased base deficit compared to the control groups (Fig. 1). Mortality in the TS30 group was 10%, compared to 30% in the TS90 shock group (*p* = 0.58). All mortality was due to trauma and shock. In the TS30 group mortality occurred 15 min after randomisation. In the TS90 group mortality occurred 40, 80 and 90 min after randomisation. All mice in the control groups survived (Table 1).

### Coagulation

**Fig. 2:**
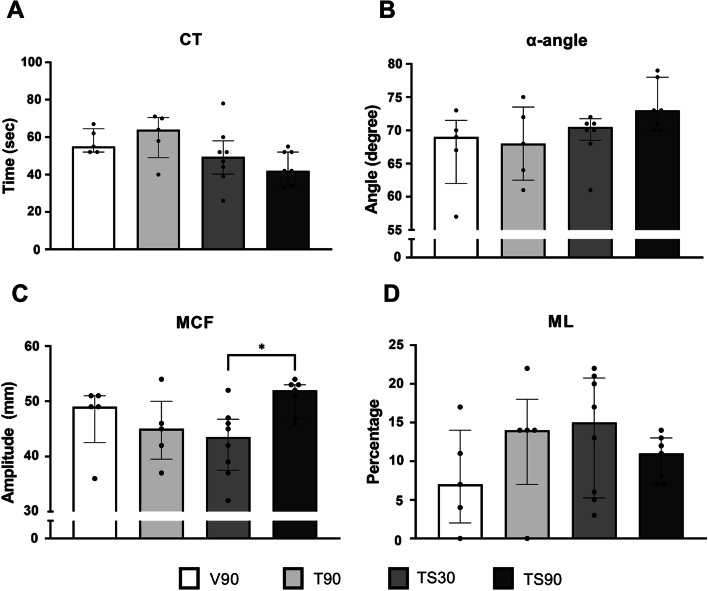
Effect of shock duration on ROTEM parameters. Data are presented as median with interquartile range. **A** EXTEM clotting time. **B** EXTEM α-angle. **C** EXTEM maximum clot firmness. **D** EXTEM maximum lysis. V90 = 90 min ventilation, T90 = trauma + 90 min ventilation, TS30 = trauma + 30 min shock, TS30 = trauma + 90 min shock. *p < 0.05 between groups

Ex-tem clotting time and alpha angle were not significantly different between groups (Fig. 2). However, maximum clotting amplitude was significantly decreased in the TS30 compared to TS90, *p* = 0.04 (Fig. 2). Median max lysis and D-dimer levels did not differ significantly between groups (Fig. 2; Table 2).

**Table 1:** Effect of shock on arterial blood gas analysis

	V90	T90	TS30	TS90
pH	7.31 (7.20–7.34)	7.27 (7.15–7.31)	7.23 (7.21–7.28)	7.08 (7.01–7.31)
pCO_2_ (mmHg)	34.0 (32.7–49.1)	40.6 (31.2–48.4)	36.2 (25.7–39.4)	38.5 (30.1–51.7)
pO_2_ (mmHg)	158.7 (106.5–194.6)	168.7 (138.0–194.0)	195.9 (179.4–214.4)	182.9 (140.7–207.8)
HCO_3_ (mM)	17.4 (16.0–19.2)	16.9 (14.5–18.1)	15.2 (10.7–17.0)	13.7 (10.3–16.6)
sO_2_ (%)	98.0 (96.1–98.3)	97.7 (96.3–98.4)	98.0 (97.3–98.5)	96.8 (95.5–98.0)
Na^+^ (mM)	146.7 (143.1–147.4)	142.4 (141.8–144.8)	143.2 (140.1–144.0)	143.0 (140.8–143.6)
K^+^ (mM)	5.8 (5.5–6.4)	6.5 (6.4–7.0)	6.5 (6.2–6.9)	7.0 (5.8–8.1)
Ca^2+^ (mM)	0.96 (0.88–1.03)	1.01 (0.93–1.03)	1.12 (1.07–1.15)	1.10 (1.07–1.22)
Glucose (mM)	6.9 (6.1–9.4)	8.0 (6.3–10.8)	9.3 (8.0–11.2)	7.7 (5.4–10.6)
Lactate (mM)	3.54 (2.73–4.03)	3.46 (3.05–3.98)	4.48 (3.26–7.25)	6.14 (4.15–9.12)

Data are presented as median with interquartile range. V90 = 90 min ventilation, T90 = trauma + 90 min ventilation, TS30 = trauma + 30 min shock, TS30 = trauma + 90 min shock

**Table 2:** Blood counts, coagulation and organ oedema

	V90	T90	TS30	TS90
Haemoglobin (mM)	8.5 (8.1–8.8)	8.4 (8.0–9.6)	7.1 (6.7–7.8)	7.1 (6.7–7.5)
Haematocrit (%)	40 (39–42)	40 (38–46)	34 (32–37)	34 (32–36)
Leukocytes (× 10^9^/l)	1.35 (0.93–1.63)	2.30 (1.95–3.55)	1.90 (1.50–3.20)	2.05 (1.23–2.83)
Platelet count (× 10^9^/l)	791 (711–942)	855 (804–875)	655 (299–799)	693 (533–784)
d-dimer (ng/ml)	1535 (1071–1595)	1060 (902–1362)	1067 (848–1278)	920 (865–938)
Organ wet/dry ratios				
Lung	3.9 (3.6–4.3)	3.9 (3.3–4.7)	4.0 (3.7–4.5)	4.1 (3.4–5.0)
Kidney	3.5 (3.4–3.8)	3.3 (3.2–3.6)	3.6 (3.4–3.8)	3.6 (3.5–3.7)
Liver	3.3 (3.2–3.4)	3.3 (3.1–3.4)	3.2 (3.1–3.3)	3.4 (3.1–3.5)

Data are presented as median with interquartile range. V90 = 90 min ventilation, T90 = trauma + 90 min ventilation, TS30 = trauma + 30 min shock, TS30 = trauma + 90 min shock

### Organ oedema

Trauma and shock did not result in significant differences in lung, kidney and liver wet/dry ratios, compared to ventilation and trauma controls (Table 2). Shock duration did not significantly influence organ oedema.

## Discussion

In this murine model of trauma and shock, we showed that short duration of shock is associated with more hypocoagulable characteristics compared to longer duration of shock.

Previous research shows that hypocoagulability is present as early as minutes after traumatic injury [2, 16]. Both the severity of tissue injury as well as the presence of shock worsens TIC [2]. Furthermore, shock and hypoperfusion are major contributors to the release of tissue plasminogen activator (tPA), converting plasminogen into plasmin, resulting in hyperfibrinolysis after trauma [17, 18]. In our model, mean values of maximum lysis and d-dimer levels did not significantly differ between groups. This could be explained by the different fibrinolytic system in mice compared to humans (i.e., shorter tPA half-life, clots are more resistant to endogenous breakdown). [19].

Our main finding, that persistence of shock reduces hypocoagulable characteristics, was contrary to our hypothesis and may seem counterintuitive. However, various studies have shown that a transition from hypocoagulable to hypercoagulable characteristics can occur early after trauma [12–14, 20]. This early shift might be explained by the increasing presence of circulating pro-coagulant platelets, exhaustion of anti-coagulant pathways and fibrinolytic shutdown [9, 11, 21, 22]. In addition, studies show that minimal amounts of coagulation factors are required for relatively normal thrombin generation [23], which could explain why thrombin generation can be increased after trauma [10]. The effect we observed could also be inflammation-induced, as pro-inflammatory pathways are tightly linked with hypercoagulability and thrombosis. [24, 25].

Our results add to the existing literature by showing that a reduction in hypocoagulability can occur early after trauma and is influenced by shock duration. Of note, the increased clotting amplitude after 90 min of shock was driven by endogenous responses, as animals did not receive treatment.

Our findings of the effect of shock duration on TIC characteristics may have several implications. Our results underline the importance of timing of treatment, as TIC characteristics change over time. Benefits of an early aggressive approach have been shown in trials investing early transfusion of blood components, as well as tranexamic acid [27, 28]. With persistence of shock, targeting dysfunctional platelets and immunomodulation may convey benefits for the severely injured trauma patient. However, these aspects of trauma-induced shock and coagulopathy need further explorations.

There are limitations to this study. Our model of traumatic shock consists of traumatic injury in combination with controlled blood withdrawals. Although the abdominal trauma results in bleeding, it is unlikely that mice continue to bleed excessively during the shock period. This means that after the blood withdrawals a relatively stable state ensues, which differs somewhat form the trauma patient with uncontrolled bleeding. Although we found decreased clot strength in the TS30 group compared to the TS90 group, both of these groups did not differ significantly from the control groups. This might be attributable to the smaller samples size in the control groups, decreasing the chance of detecting differences between the control groups and the shock groups. In addition, since more mice died after 90 min shock compared to 30 min shock, survival bias might explain part of the observed effect. The mice that died during the experiments were not included in our analysis, lowering the sample size and, therefore, the power to detect a difference in the primary outcome. Finally, we have not dissected the coagulation pathways explaining the difference in maximum clot firmness in the ROTEM. We can, therefore, only speculate about the mechanisms.

In conclusion, hypocoagulability is part of early endogenous TIC and alters with prolonged shock duration. More research is needed to unravel the mechanisms behind this shift to develop more targeted treatments for trauma-induced shock.

## Supplementary Information

**Additional file 1: Fig. S1. **Experiment overview.

## Data Availability

All data generated or analysed during this study are included in this published article. The data sets used and/or analysed during the current study are available from the corresponding author on reasonable request.

## Abbreviations

CT: Clotting time
ELISA: Enzyme linked immune sorbent assay
FiO_2_: Fractional inspired oxygen
MAP: Mean arterial pressure
MCF: Maximum clot firmness
ML: Maximum lysis
ROTEM: Rotational thromboelastometry
TIC: Trauma-induced coagulopathy
