# Supplementary material for: The effect of shock duration on trauma-induced coagulopathy in a murine model
Source: Intensive Care Med Exp. 2022 Jan 7;10:1. doi: 10.1186/s40635-021-00428-1 (PMC8738789; doi:10.1186/s40635-021-00428-1)
Supplement: Supplementary file 1 — Additional file 1: Fig. S1. Experiment overview. [file 40635_2021_428_MOESM1_ESM.docx]

**Supplemental figure 1. Experiment overview**

**
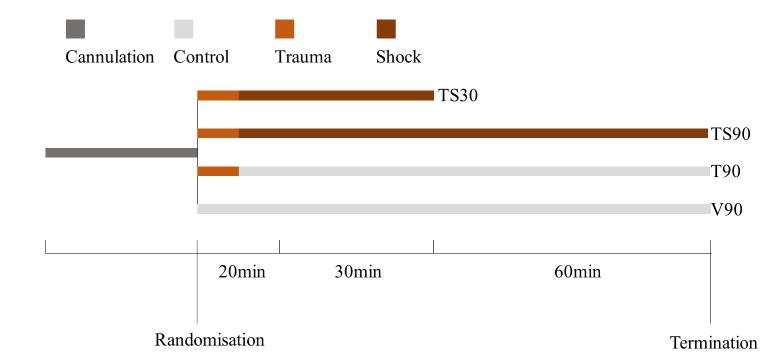
**

*After the cannulation period, mice were randomized into four experimental groups: Ventilation only (V90), Trauma only (T90), Trauma and 30min shock (TS30) and trauma and 90min shock (TS90). Trauma consisted of bilateral lower limb fractures in combination with laparotomy followed by crush injury to the small intestine and liver. The trauma procedure took approximately 10 minutes. Shock was induced by a controlled blood withdraw before and after trauma, until a MAP of 25-30mmHg was achieved. The blood withdraws took approximately 10 minutes. Twenty minutes after randomisation mice were mechanically ventilated for an additional 90min (V90, T90, TS90) or 30min (TS30), after which the mice were sacrificed.*
